# A case of tuberculous meningitis triggered by long-term use of corticosteroids drugs

**DOI:** 10.1097/MS9.0000000000003310

**Published:** 2025-04-22

**Authors:** Noha Zaki, Osman Mohamed, Taha Eltayeb, Dalya Mohammed

**Affiliations:** a Internal Medicine Department, Sea Port Co-operation Hospital, Port-Sudan, Sudan; b Internal Medicine Department, Red Sea University, Port-Sudan, Sudan

**Keywords:** central nervous system infection, corticosteroid, immunosuppression, tuberculosis, tuberculous meningitis

## Abstract

**Introduction and importance:**

Tuberculous meningitis (TBM), caused by *Mycobacterium tuberculosis*, is a severe central nervous system infection prevalent in areas with a high incidence of tuberculosis. Dissemination frequently takes place among children and young adults within particular regions.

**Case presentation:**

A 25-year-old woman from East Sudan presented with headache, neck pain, fever, anxiety, and hemodynamic instability. Despite no prior history of tuberculosis or known contact with affected individuals, her diagnosis of TBM was confirmed based on clinical presentation, radiological findings (notably leptomeningeal enhancement), and a positive response to anti-tuberculosis therapy.

**Clinical Discussion:**

This case highlights the importance of immunosuppressive therapy as a risk factor, as the patient had been on a prolonged course of corticosteroids (dexamethasone) at a dose of 15 mg/day for 6 months. This atypical presentation emphasizes the relevance of TBM in patients with prolonged symptoms of meningitis that do not respond to standard treatment, especially those with a history of immunocompromising diseases or drugs.

**Conclusion:**

This case demonstrates the serious implications of inappropriate corticosteroid usage, as well as the crucial importance of early diagnosis and treatment of TBM to avoid life-threatening complications. It serves as a reminder of the significance of remaining vigilant for TBM in areas where tuberculosis is endemic.

## Introduction

Tuberculosis meningitis (TBM), tuberculoma, and spinal arachnoiditis are the main diseases of the central nervous system (CNS) caused by *Mycobacterium tuberculosis*. TBM has a significant mortality risk, impacting 1–5% of patients diagnosed with pulmonary tuberculosis[1]. Its diagnosis relies on clinical presentation, which sometimes being atypically, supplemented by cerebrospinal fluid (CSF) analysis and advanced imaging techniques, such as computed tomography (CT) or magnetic resonance imaging (MRI)^[^2,3^]^.

Detecting CNS infections in immunocompromised individuals, such as those with cancer, organ transplants, or long-term steroid treatment, presents a unique challenge. Corticosteroids, in particular, can obscure clinical and radiographic findings, often resulting in reduced or absent contrast enhancement, further complicating diagnosis[4]. This complexity is exacerbated in resource-limited settings, where access to comprehensive evaluations is often constrained. Under such circumstances, a thorough medical history and high clinical suspicion are essential for accurate diagnosis[2]. However, diagnosing TBM in a patient previously in excellent physical condition posed a significant challenge.

**HIGHLIGHTS**

- This case highlights the role of corticosteroid-induced immunosuppression in the development of tuberculosis meningitis (TBM) and the consequent diagnostic dilemmas due to its nonspecific presentation.
- An atypical presentation of TBM in an immunocompetent individual during long-term use of corticosteroids emphasizes the importance of considering TBM in the absence of classical risk factors.
- The patient’s remarkable earliest response to anti-tuberculous medication underscores the significance of early clinical suspicion in high-risk populations to improve outcomes.

## Case presentation

**Figure 1.**
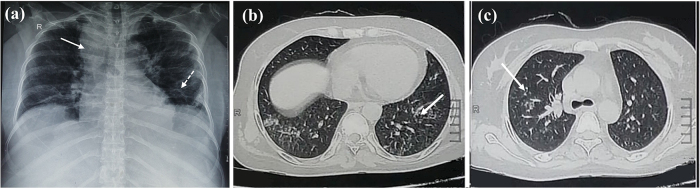
**(a)** Chest x-ray shows the right upper lobe nodules (solid white arrow) and reticulonodular shadowing mainly in the left lower lobe (white dotted arrow). **(b)** CT chest showing bilateral reticulonodular shadowing mainly in the left lower lobe, represented with white arrows. **(c)** CT chest demonstrates ground glass opacities, mainly in the right upper lobe, represented with a white arrow.

**Figure 2.**
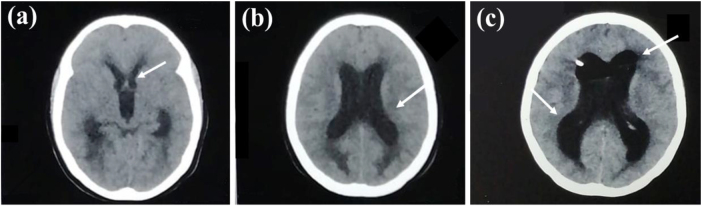
CT brain shows **(a)** leptomeningeal enhancement and widening of temporal horns with **(b)** mild enlarged lateral and third ventricles. **(c)** CT Brain done almost one month after the previous one illustrates dilated temporal horns and extensively enlarged third and lateral ventricles.

A 25-year-old woman from East Sudan with no significant medical history aside from aiming to get weight gain, the patient had been taking 15 mg/day of dexamethasone for 6 months, purchased without a prescription for cosmetic reasons. This extended, uncontrolled corticosteroid usage remarkably caused immunosuppression, which increases the patient’s risk of infections, including TBM; presented to the hospital with a persistent and severe headache for 2 weeks, experienced neck pain, high fever (39°C), anxiety, nausea, vomiting for 2 days, confusion, low blood pressure (90/60 mmHg), rapid pulse (110 bpm), increased respiratory rate (25 breaths/min), normal responsive pupils, neck stiffness, hyperreflexia, and epigastric tenderness upon examination. To rule out any underlying gastrointestinal pathology, an abdominal ultrasound was performed, which revealed no abnormalities. By day 2 post-admission, there was a progressive drop in the level of consciousness, seizures, and right upper motor neuron facial nerve palsy. Laboratory investigations revealed leukocytosis (12.6 × 10^9^/L) with predominant neutrophilia (6.87 × 10^9^/L), lymphocytes (4.8 × 10^9^/L) hemoglobin of 11.9 g/dL, elevated C-reactive protein (45 mg/L), and lactate dehydrogenase (537 U/L) as shown in Table 1. Renal and liver function tests were unremarkable. Tuberculosis (GeneXpert), coronavirus (PCR), and malaria (blood films) tests were negative. Chest X-ray (Fig. 1a) shows multiple nodules with reticulonodular opacities. A brain CT indicated bilateral leptomeningeal enhancement (Fig. 2). Although the patient’s lymphocyte count was within the normal range (4.8 × 10^9^/L), the history of prolonged corticosteroid use strongly suggests impaired cell-mediated immunity. While advanced immune function tests were not available, the clinical context supports the hypothesis of corticosteroid-induced immunosuppression as a contributing factor to the development of TBM.

**Table 1:** laboratory investigation results

Test	Observed Value	Reference value
CBC		
TWBCs	12.6 × 10^9^/L	(4.0–11.0) × 10^9^/L
Neutrophils	6.87 × 10^9^/L	(1.8–6.8) × 10^9^/L
Lymphocytes	4.8 × 10^9^/L	(1.2–4.8) × 10^9^\l
HB	11.99 g/dL	(11.0–16.0) g/dL
PLTs	398 × 10^9^/L	(150–400) × 10^9^/L
Others		
CRP	45 mg/L	Less than 5 mg/L (Negative)
LDH	537 U/L	140–280 U/L
LFT (After one month of starting anti-tuberculous treatment)		
Total Bilirubin	2.1 µmol/L	(Up to 1.1) µmol/L
Direct Bilirubin	1.43 µmol/L	(Up to 0.3) µmol/L
ALT	209 IU/L	(Up to 41) IU/L
AST	122 IU/L	(Up to 40) IU/L

CBC, complete blood count; Hb, hemoglobin; TWBCs, total white blood cells. PLTs, Platelets counts, LFT, liver function test; ALT, alanine transaminase; AST, aspartate transaminase; CRP, C- reactive protein; LDH, lactate dehydrogenase.

Given the concern for potential immunosuppression, the patient underwent HIV screening, which returned negative, effectively ruling out HIV/AIDS-related immunosuppression as an underlying cause.

Initial CT brain imaging revealed modest enlargement of the lateral and third ventricles, bilateral leptomeningeal enhancement, and broadening of the temporal horns, which were consistent with probable TBM, developing hydrocephalus, and elevated ICP (Fig. 2). A CT chest was also done (Fig. 1b,c). Given these radiological signs of increased ICP, lumbar puncture (LP) was withheld due to the significant risk of brain herniation.

The patient had initially been put on high-dose ceftriaxone, anticonvulsants, and dexamethasone. However, there was a gradual poor response to treatment; thus, a strong suspicion of TBM led to the start of anti-tuberculous therapy. Empirical anti-tuberculous therapy was initiated promptly following national tuberculosis treatment guidelines. The patient commenced treatment with the standard four-drug regimen (HRZE), which includes rifampicin (10 mg/kg), isoniazid (5 mg/kg), pyrazinamide (25 mg/kg), ethambutol (15 mg/kg) and pyridoxine. Due to the CNS involvement, adjunctive corticosteroid therapy with dexamethasone (0.4 mg/kg/day) was implemented to reduce inflammation-related complications. The patient’s consciousness progressively improved, though she remained aphasic.

Subsequent imaging of the CT brain demonstrated ventricular enlargement and temporal horn lesions (Fig. 2c). Due to limited resources, the patient was referred to another facility for MRI, which confirmed active communicating hydrocephalus with periventricular edema, diffuse supra- and infratentorial meningeal enhancement, and spinal involvement, including anterior wedging of the C3 vertebra with subligamentous enhancing collection. The radiological impression strongly suggested TBM with increased ICP, prompting immediate neurosurgical intervention. A ventriculoperitoneal shunt was inserted, resulting in clinical improvement.

In the absence of CSF analysis, the diagnosis of TBM relied on a combination of clinical presentation, radiological findings, and epidemiological risk factors. The therapeutic decision-making process adhered to established clinical guidelines and evidence-based practices to enhance patient outcomes and reduce risks linked to invasive procedures in cases of elevated ICP. This case highlights the critical importance of prioritizing patient safety with elevated ICP. It underscores the role of advanced imaging, such as MRI, in diagnosing TBM when LP is contraindicated.

Additional diagnostic tests for tuberculosis, such as Mantoux testing, serum IGRA, and sputum analysis for acid-fast bacilli, were considered but not performed initially due to the urgency of the clinical presentation and the need for immediate neurosurgical intervention.

One month later, the patient developed jaundice and elevated liver enzymes (AST 122 IU/L, ALT 209 IU/L, total bilirubin 2.1 µmol/L, direct bilirubin 1.43 µmol/L) as shown in Table 1. The patient’s AST and ALT levels were elevated by five times the upper limit of normal, which is classified as mild to moderate elevation. Despite intense counseling about the importance of adherence to anti-tubercular therapy, the patient and family discontinued therapy. Deterioration occurred, ultimately resulting in her death after three months without further medical care. This particular case calls to attention the serious consequences of delayed or incomplete treatment of TBM. It emphasizes the imperative to strictly follow anti-tuberculous therapy, particularly for immunocompromised patients.

A thorough differential diagnosis was carried out. Based on clinical presentation and laboratory results, TBM became the most likely diagnosis considering the extended corticosteroid usage, which raises infection susceptibility. We carefully assessed alternative sources of CNS involvement, including viral, fungal, and autoimmune diseases. Lack of typical neurological and systemic symptoms led to the ruling out of viral meningoencephalitis (e.g., HSV, VZV). Given the epidemiological background and absence of systematic fungal signs, Cryptococcal meningitis was considered unlikely. Although it is a vital diagnostic tool for cryptococcal meningitis, serum cryptococcal antigen testing was inaccessible in our setting. Although neurosarcoidosis and other autoimmune etiologies were thought about, they were discounted because the patient responded well to anti-tuberculous treatment and lacked systemic autoimmune features. Nevertheless, the patient’s clinical trajectory and treatment response supported TBM as the main diagnosis, therefore underlining the need of a comprehensive clinical approach especially in settings with limited resources.

## Discussion

TBM is an acute and lethal infection of the CNS characterized by unclear incidence and mortality figures, highlighting the difficulties in generating epidemiological data on the disease owing to the lack of precise laboratory and clinical diagnostic criteria. In Sudan, TBM is still a major public health concern, especially in resource-constrained environments where delayed diagnosis and restricted access to modern diagnostic equipment led to considerable morbidity and death. Recent epidemiological data indicates that Sudan has a significant tuberculosis burden, with extrapulmonary tuberculosis at around 22% and TBM at around 2%[5]. Early imaging and clinical suspicion are essential in immunosuppression; thus, the clinical presentation of TBM in our patient fits the results from similar case studies^[^6,7^]^. These findings and our case highlight the necessity of more awareness and enhanced diagnostic tools in areas with high tuberculous frequency. Although this entity is rare in an immunocompetent host, it occurs more frequently in immunocompromised individuals.

The immunosuppressive effects of corticosteroids, such as dexamethasone, exacerbate the risk of infection. While concurrently encouraging the development of anti-inflammatory mediators, corticosteroids primarily function as immunosuppressors by blocking the transcription of pro-inflammatory cytokines like interleukin-1 (IL-1), interleukin-6 (IL-6) and tumor necrosis factor-alpha (TNF-α). This imbalance compromises cell-mediated immunity, especially the roles of macrophages and CD4+ T-cells, which are essential for Mycobacterium Tuberculosis control^[^8,9^]^. A study emphasizes prolonged corticosteroid use – as seen in our patient – has been demonstrated to raise the chance of developing tuberculosis [10]. Moreover, clinical studies show that although supplementary glucocorticoid treatment can lower mortality in TBM by reducing inflammation and avoiding complications such as hydrocephalus, its time and dosage must be precisely adjusted to minimize aggravation of immunosuppression. As observed in a study, early administration of dexamethasone in patients with TBM greatly improved outcomes, yet extended usage beyond 6 to 8 weeks was linked to higher secondary infection risk[11]. These results highlight the careful equilibrium in the control of TBM between immunosuppressive hazards and therapeutic advantages. Prolonged corticosteroid therapy, even at doses considered low to moderate (e.g., 10–15 mg/day of prednisone or its equivalent), significantly increases the susceptibility to infections, with a marked increase in risk observed after three months of continuous use^[^12–14^]^.

Moreover, in regions in which over-the-counter access is somewhat ubiquitous, the non-prescription use of corticosteroids for cosmetic purposes generates public health problems. Extended self-administration of dexamethasone, particularly at levels of 10–15 mg/day, has been shown to reduce both innate and adaptive immunity, therefore raising the risk of opportunistic infections, including TBM. For example, a study investigation revealed that even short corticosteroid usage was linked with a notable rise in the likelihood of infections, especially when regulatory control is inadequate[15].

Diagnosing TBM is challenging due mainly to its tremendous potential to imitate other forms of meningitis. The performance of diagnostic tools available for confirmation is insufficient, and current methods of diagnosis leave much to be considered in terms of their sensitivity and specificity. Due recognition of factors on patient history, endemicity for tuberculosis, and analysis of suitable LP findings are the most useful diagnostic clues. The diagnosis of TBM requires a demonstration of mycobacterium tuberculosis in the CSF, but such a demonstration reflects diagnosis after several days. Hence, we may still often be left with no definitive biochemical test. The LP, which enables the drawing of CSF for laboratory evaluation, is difficult, especially in a patient with TBM and elevated ICP.

Radiological imaging forms the cornerstone. CT helps visualize classic signs such as basal meningeal enhancement, infarctions, and hydrocephalus. MRI is more sensitive and specific, thereby better at detecting brainstem involvement and neurological complications^[^16–18^]^.

This scenario emphasizes various crucial facets of TBM. The patient’s Eastern Sudanese background, which is known for its high tuberculosis rates, along with the lengthy and intensive usage of dexamethasone, posed substantial risk factors for serious infections such as TBM. Significantly, the patient lacked any previous record of immunosuppression, primary tuberculosis, or documented contact with persons having tuberculosis. This highlights the significance of corticosteroid-induced immunosuppression in the development of TBM. The absence of response to empirical treatment for bacterial meningitis, coupled with significant improvements on anti-tuberculous therapy, highlights the need to consider TBM as a potential diagnosis, especially among high-risk groups. Although the main emphasis of this case was TBM, it is crucial to recognize the rising challenges of antibiotic resistance in bacterial meningitis, especially with organisms like *Streptococcus pneumoniae* and *Neisseria meningitidis*. Rising reports of ceftriaxone resistance in areas with substantial antibiotic use call for the use of other drugs, notably rifampicin, which has shown effectiveness against these pathogens. For instance, research underlined the value of rifampicin as an additional therapy in ceftriaxone-resistant bacterial meningitis, especially in circumstances where immediate confirmation is not possible[19]This emphasizes the need to consider local resistance patterns before starting empirical treatment for meningitis, particularly in situations with limited resources.

In this case, early improvement of the patient following the initiation of anti-tuberculous therapy provides further confirmation of the diagnosis, thus indicating a need for great clinical suspicion, and timely intervention should be taken in similar cases to avoid fatal consequences. Efforts were made to advise the patient and her family that continued therapy was necessary; their uninformed choice to withdraw treatment led to death. The case emphasizes the necessity of effective patient education and adherence to treatment regimens.

This case holds great importance; it highlights the crucial importance of being aware of TBM, especially in patients who are undergoing long-term corticosteroid therapy and reside in or originate from regions with a high prevalence of tuberculosis. Furthermore, it demonstrates the difficulties in diagnosing and considering TBM when conventional treatments are ineffective. It provides valuable insight into the diagnostic process and clinical decision-making, backed by extensive patient history and examination results. However, its limitations include the inherent constraints of single-patient data, which limit the generalizability of findings, and the lack of improved diagnostic techniques, which could increase understanding of disease causes and diagnostic accuracy.

## Conclusion

TBM is a major cause of CNS tuberculosis mortality, often underdiagnosed due to overlapping symptoms. Early detection relies on thorough clinical evaluation and a high index of suspicion, especially in immunocompromised individuals.

## Data Availability

The dataset and/or analyzed during the study is available from the corresponding author upon reasonable request.
